# Perturbation of microbiota in one-day old broiler chickens with antibiotic for 24 hours negatively affects intestinal immune development

**DOI:** 10.1186/s12864-017-3625-6

**Published:** 2017-03-20

**Authors:** Dirkjan Schokker, Alfons J. M. Jansman, Gosse Veninga, Naomi de Bruin, Stephanie A. Vastenhouw, Freddy M. de Bree, Alex Bossers, Johanna M. J. Rebel, Mari A. Smits

**Affiliations:** 1 Wageningen Livestock Research, Postbus 338, 6700 AH Wageningen, The Netherlands; 2 Cobb Europe BV, Boxmeer, The Netherlands; 3 Gezondheidsdienst voor Dieren, Deventer, The Netherlands; 4 Wageningen Bioveterinary Research, Lelystad, The Netherlands

**Keywords:** Chicken, Gut, Microbiota, Gene expression, Immune development

## Abstract

**Background:**

Gut microbial colonization and development of immune competence are intertwined and are influenced by early-life nutritional, environmental, and management factors. Perturbation of the gut microbiome at young age affects the crosstalk between intestinal bacteria and host cells of the intestinal mucosa.

**Results:**

We investigated the effect of a perturbation of the normal early life microbial colonization of the jejunum in 1-day old chickens. Perturbation was induced by administering 0.8 mg amoxicillin per bird per day) via the drinking water for a period of 24 h. Effects of the perturbation were measured by 16S rRNA profiling of the microbiome and whole genome gene expression analysis. In parallel to what has been observed for other animal species, we hypothesized that such an intervention may have negative impact on immune development.

Trends were observed in changes of the composition and diversity of the microbiome when comparing antibiotic treated birds with their controls. in the jejunum, the expression of numerous genes changed, which potentially leads to changes in biological activities of the small intestinal mucosa. Validation of the predicted functional changes was performed by staining immune cells in the small intestinal mucosa and a reduction in the number of macrophage-like (KUL01^+^) cells was observed due to a direct or indirect effect of the antibiotic treatment. We provide evidence that a short, early life antibiotic treatment affects both the intestinal microbiota (temporarily) and mucosal gene expression over a period of 2 weeks.

**Conclusion:**

These results underscore the importance of early life microbial colonization of the gut in relation to immune development and the necessity to explore the capabilities of a variety of early life dietary and/or environmental factors to modulate the programming for immune competence in broilers.

**Electronic supplementary material:**

The online version of this article (doi:10.1186/s12864-017-3625-6) contains supplementary material, which is available to authorized users.

## Background

Nutrient intake and immune homeostasis are important aspects for chicken health. These aspects are influenced by many different factors, for instance by the composition and diversity of the resident intestinal microbial population, by feed composition and by host genetics [1, 2]. In research, already quite a lot of attention is given to genetics, housing, and diet, in order to generate vital broilers, regarding their performance and immune competence. However, little attention was given so far to the role of microbiota for healthy broilers [3, 4]. The colonization of the gut by microbiota in young animals occurs simultaneously with the development of the gut tissues [3, 5–7]. Later in life, interactions between microbiota and mucosal host cells influence the functioning of the gut system. This intimate interplay affects digestion, maintenance of gut barrier integrity, and immune homeostasis [8]. After hatch, the immune system develops rapidly and also this development heavily influenced by the early life microbial colonization of the gut. Dietary interventions at young age, such as the usage of (pre)starter feeds, prebiotics, probiotics and antibiotics, are regarded to affect the crosstalk between microbiota and host mucosal cells in the intestinal tract, which may result in a change of immune development [8–11]. During the first weeks of life different categories of immunological processes have been identified in broilers [12–14]. Based on spatio-temporal gene expression profiles, the following sequential order for immune related processes have been reported: 1) innate development and influx of immune cells; 2) immune differentiation and specialization; and 3) maturation and immune regulation [14].

For pigs, it has already been shown that administration of antibiotics during early life, day 4 or day 28 of age, leads to altered composition and diversity of microbiota in the gut [10, 11, 15]. These perturbations also affected the expression of numerous immune related genes in the gut mucosal tissue for a longer period of time (up till 56 days after treatment). This indicated an important role for the early gut microbial colonizers for the development and/or programming of the mucosal immune system. In addition, studies in mice and humans show that modulating the microbial colonization in early life by antibiotics, can lead to higher risks for developing immunity based disorders, such as asthma and allergy [2, 16, 17]. Therefore, it is worthwhile to investigate the importance of early life microbial colonization in chicken on immune status, which is relevant for the development of early life nutritional strategies to produce vital broiler chicks, regarding their performance and immune competence. In parallel to what has been observed for other animal species, we hypothesized that an early life intervention with an antibiotic may have negative effects on immune development [2, 15, 18, 19]. We compared a non-disturbed microbial colonization profile of control chicken with a disturbed microbial colonization profile of chicken that received the antibiotic (a dose of approximately 0.8 mg amoxicillin [=19 mg/kg body weight/day]) at day 1 after hatch. Amoxicillin is a broad-spectrum antibiotic of the penicillin family. Amoxicillin is active against some Gram-negative and most Gram-positive bacteria. However, amoxicillin is not effective against beta-lactamase producing organisms. For poultry this antibiotic is used in Europe, but not in the United States. In Europe amoxicillin is used for the treatment and prevention of bacterial infections. In poultry this includes alimentary, urogenital and respiratory tracts infections [20]. The recommended dose (by the manufacturer) is 10 to 20 mg of the product per kg of body weight (i.e. 8–16 mg/kg amoxicillin trihydrate) per day, and should be administered via the drinking water. According to the recommendations of the manufacturer, the product should be administered for 3–5 consecutive days to be effective in preventing clinical signs of disease. However, in this study we used healthy animals and therefore we administered the amoxicillin only once in order to perturb the intestinal microbiome during early-life.

The objective of this study was to investigate the impact of a short-term (24 h) perturbation of the normal early life microbial colonization of the gut of broiler chickens with an oral dose of amoxicillin on the diversity and composition of the intestinal microbiota over a period of 2 weeks. In addition, we investigated the impact of the perturbation on the host intestinal gene expression and the number and identity of immune cells in the gut mucosal tissue over the same period of time. To find out whether the microbiota and host gene expression were altered by the antibiotic treatment, we collected jejunal samples at three time-points: day 1 after hatch (prior to antibiotic administration); day 5; and day 14. From the jejunum the luminal content was taken to perform in depth 16S rRNA sequencing of the resident microbiota and jejunal tissue was taken to perform genome wide gene expression analyses and to characterize and quantify intestinal immune cells.

## Methods

### Housing, diet, and experimental design

In this experiment 1-day-old chickens (Cobb500, both males and females) with an average body weight of approximately 42 g at hatch, were housed in a floor pen system with litter (wood shavings). Chickens had *ad libitum* access to crumble feed and water. All treatment groups and pens received feed from the same batch. For each of the treatments, control or antibiotic, eight pens were used. After the treatments chicks were housed on fresh litter.

Representative feed samples were used for chemical analyses, including the Kjeldahl method for nitrogen determination and ashing at 550° Celsius to determine the amount of ash (Table 1). We assumed that chickens will consume approximately 0.012 L water the first day. The antibiotic treatment (24 h) consisted of 0.067 g amoxicillin per litre drinking water, which corresponds to approximately 0.8 mg amoxicillin per bird per day and to 19 mg amoxicillin per kg body weight per day, as advised by the manufacture.

**Table 1:** Composition of the broiler diet

	broiler starter diet
Ingredient composition	(% inclusion)
Maize	35.03
Soybean meal	30
Wheat	27.6
Premix^a^	2
Soybean oil	1.92
Palm oil	1
Chalk	0.7
Monocalcium phosphate	0.66
DL-methionine	0.26
Sodium bicarbonate	0.23
Lysine HCl	0.22
NaCl	0.19
L-threonine	0.05
Enzyme (NSP degrading ^b^)	0.02
Enzyme (Phytase ^c^)	0.01
Calculated composition	g/kg
Crude protein	209
Ether extract	56
Crude fibre	24.6
Ash	51.8
Starch (amylase)	388.6
Ca	7.8
P	5.2
Cl	1.7
Na	1.6
K	8.6
6-phytase (PU ^d^/kg)	500
Digestible lysine	11.2
Digestible methionine	5.4
Digestible methionine + cysteine	8.3
Digestible threonine	6.9
Digestible tryptophan	2.2
Digestible isoleucine	7.7
Digestible valine	8.3
Digestible arginine	12.5
Digestible glycine + serine	16
Absorbable phosphorus	4.2
Analysed composition (g/kg)	
ME (kcal/kg)	2900
Dry matter	873.4
Crude ash	50.6
Crude protein	201.1
Crude fat	60.5
Crude fibre	29.5
Starch	375.5
Calcium	7.7
Phosphorus	5.2

^a^Premix supplied per kg of feed. Vitamin A, 12 500 (internation unit (IU); Vitamin D3 3500 IU; Vitamin Hy-D, 0.025 mg; Vitamin E, 100 IU; Vitamin K3, 4 mg; Vitamin B1 4 mg; Vitamin B2 9 mg; Pantothenic acid 20 mg; Niacinamide, 70 mg; Vitamin B6, 6 mg; Folate, 1.5 mg; Vitamin B12, 30 μg; Biotine 250, μg; Betaine, 150 mg; L-Carnitine, 30 mg; Fe, 50.0 mg; I, 2.0 mg; Cu, 14.0 mg; Mn, 55 mg; Zn, 100 mg; Se, 0.3 mg

^b^Hostazyme X®

^c^Phyzyme® XP 5000 L

^d^PU, phytase units

At three time-points during the study (days 1, 5, and 14) birds were sacrificed for tissue sampling. Birds were first anesthetised, followed by decapitation, the animal ethics board advised and approved this procedure [21, 22]. At day 1, before the antibiotic treatment, 80 chickens were sacrificed. At days 5 and 14, 160 chickens were sacrificed, 80 control and 80 antibiotic treated chickens, respectively. For gene expression studies the samples were taken from mid-jejunum (whole tissue; 2–3 cm) and snap frozen in liquid nitrogen. The adjacent anterior part (approximately 5 cm) was used for sampling the jejunal microbiota (by extracting the luminal content and directly deposit this into a tube) and also snap frozen in liquid nitrogen. Lastly, the adjacent posterior part (whole tissue; 2 cm) was snap frozen in liquid nitrogen and used for immunohistochemistry. All the samples were stored at -80 °C until further analyses.

**Fig. 1:**
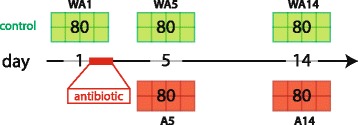
Schematic representation of experimental design. Eighty birds were sacrificed at day 1, 5, 14 for control birds (without antibiotic), WA1, WA5, and WA14 respectively, and at day 5 and 14 for antibiotic treated birds, A5 and A14 respectively. In total there are 5 treatment-day combinations consisting of up to 8 points, where each point represents 1 pool consisting of 10 chicken. The antibiotic, amoxicillin, was administrated for 1 day, starting at day 1 and lasting for 24 h, via the drinking water. At all sampling days, the jejunal segment was taken, for sequencing of the luminal microbiota, host gene expression and immunohistochemical staining of different immune cells

Subsequently, for each combination of time-point and treatment (5 groups) RNA or DNA of individual chickens were pooled for further analysis. Pooling was performed per pen, because this was the unit of interest. Per time-point/treatment combination 10 RNA or DNA of individual chickens made up a pool and in total there were 8 pools (see Fig. 1). Animals were pooled, because our main focus was to get more insight into biological processes (bacterial colonization, intestinal development) at the population level.

### Performance data

The performance of the animals was defined as body weight or feed conversion ratio (FCR) per pen. Body weight was measured at days 5, 7, 14, 21, and 35. Whereas the FCR was calculated over certain time periods, namely 0–5, 0–7, 0–14, 0–21, 0–35.

### Microbiota data

For a detailed description see a previous study by Schokker et al. [23]. Briefly, digesta of mid-jejunum was collected for all birds, by gently stripping the gut segment into a plastic container, and immediately snap-frozen in liquid nitrogen and subsequently stored at -80 °C until further analysis. DNA extraction was performed followed by the V3 PCR sequencing and Quantitative Insights Into Microbial Ecology (QIIME) pipeline [24]. Statistical analysis were performed by the vegan package (http://cran.r-project.org/web/packages/vegan/) within the R environment, i.e. Shannon diversity index and Redundancy analysis (RDA). For over- and under-representation of bacterial groups a (non-parametric) Wilcoxon signed-rank test was performed on the family level, and *p*-values were converted to false discovery rate (FDR) values to correct for multiple testing. Where FDR values below 0.05 were treated as significant, and FDR values between 0.05 and 0.1 as trends.

### Transcriptomics data

For a detailed description see a previous study by Schokker et al. [23]. Briefly, total RNA was extracted from 50 to 100 mg jejunum tissue. Jejunum is the intestinal segment of interest, mainly because the jejunum is both involved in absorption of nutrients and minerals, exerts immunological activity and in early life this segment is involved in the programming of the (local) immune system [13, 14]. Each sample was handled individually and subsequently pools were made for further analyses. Labelling, Hybridization, Scanning, and Feature Extraction were all performed as recommended by Agilent Technologies. The data discussed in this publication have been deposited in NCBI’s Gene Expression Omnibus [25] and are accessible through GEO Series accession number GSE67452 (https://www.ncbi.nlm.nih.gov/geo/query/acc.cgi?acc=GSE67452). The data were analysed by using R (v3.0.2) by executing different packages, including linear models for microarray data (LIMMA) [26] and arrayQualityMetrics [27]. On the data a background correction was performed (method = “normexp” and offset = 1) with functions from the R package LIMMA [26] from Bioconductor [28]. Followed by quantile normalisation, thereafter duplicate probes (probes mapping to the same gene) were averaged (by performing the ‘avereps’ method). Subsequently, the lower percentile of probes were removed in a three-step procedure [23, 26]. Lastly, statistical and functional genomics analysis were performed. To test the differences between the experimental groups (without antibiotic and antibiotics) on both day 5 and 14, the following contrasts, A5-WA5 and A14-WA14, were generated within the LIMMA package [26]. The Database for Annotation, Visualization and Integrated Discovery (DAVID) was used to perform Functional Annotation Clustering (FAC) for the two different contrasts, i.e. A5-WA5 and A14-WA14. Up- and down-regulated genes were separately analysed.

### Immunohistochemistry

Jejunal cryosections, 8 μm thick, were stained with specific antibodies using an indirect immunoperoxidase staining method as described by Schokker et al. [29]. Briefly, slides were treated for endogenous peroxidase activity, blocked with BSA, and incubated with monoclonal antibodies against CD4^+^ cells, CD8^+^ cells, or macrophage-like cells (CT-4, 1:200; CT-8, 1:200; and KUL01, 1:50, respectively; Southern Biotech, Birmingham, AL), followed by peroxidase-conjugated rabbit anti-mouse Ig (P0161, Dako, Denmark). Peroxidase activity was detected by 3,3-diaminobenzidine, and sections were counterstained with haematoxylin. Negative controls were performed by omitting of the primary antibody. For each sample 3 to 4 mm^2^ mucosa (without muscular layers) were evaluated by 10x magnification on a bright field microscope. Subsequently, the samples were further analysed using Olympus cellSens Dimension (version 1.7.1) software. First positive-stained cells were counted, secondly these cells were averaged per time point and group, and lastly they were represented as positive-cells per tissue area (square mm). A Student’s *T*-test was performed to calculate the significance between the treatment and control on each time-point separately.

## Results

### Animal performance

Both the body weight and feed conversion ratio (FCR) were not significantly different between antibiotic treated birds and control birds. Body weights showed an increase in time, from approximately 130 g at day 5 to 2.3 kg at day 34 (Table 2). The FCR also increased over time, from approximately 0.78 at day 0–5 to about 1.66 at day 0–34 (Table 3). A FCR below 1 is possible in broilers, because the chicks also absorb the yolk sac in the first days of life [30].

**Table 2:** Body weight (g) at different time-points comparing antibiotic versus control birds

Day	WA^a^	A^b^	SEM	*p*-value*
5	132	129	1.0	0.18
7	181	188	2.9	0.25
14	501	505	6.2	0.75
21	950	960	11.9	0.69
34	2278	2276	44.2	0.98

^a,b^ *Abbreviations*: *WA* without antibiotic; *A* with antibiotic

*Student’s *t*-test

**Table 3:** Feed conversion ratio (FCR) of different time-slots of antibiotic versus control birds

Time slot	Average WA^a^	Average A^b^	SEM	*p*-value*
0–7	0.79	0.77	0.01	0.56
0–14	1.20	1.18	0.01	0.46
0–21	1.59	1.58	0.02	0.75
0–34	1.65	1.67	0.01	0.44

^a,b^ *Abbreviations*: *WA* without antibiotic; *A* with antibiotic

*Student’s *t*-test

### Microbiota analyses

To get generic insight into the microbiota and relative abundances of microbial groups over time, we selected the top 9 most abundant bacterial groups (7 defined families) over all three time-points in control birds (no antibiotics, Table 4). This shows that the most dominant families, based on their relative contribution, on day 1 were the Enterobacteriaceae (61.1%) and *Enterococcaceae* (25.9%), however for day 5 the most dominant families were the *Lactobacillaceae* (77.9%) and *Enterococcaceae* (21.7%). At day 14, again a shift in dominant families was observed when compared to the previous recorded time-point. At day 14 *Lactobacillaceae* (82.2%) and *Streptococcaceae* (8.9%) were the most dominant families.

**Table 4:** Relative abundance of major bacterial groups in the jejunum at d 1, 5 and 14

Phylum	Class	Family	WA1^a^	WA5	A5^b^	WA14	A14
Firmicutes	Bacilli	Enterococcaceae	**25.9** ^**c**^	**21.7**	**25.2**	4.9	9.9
Firmicutes	Bacilli	Lactobacillaceae	0.5	**77.9**	**74.2**	**82.2**	**70.5**
Firmicutes	Bacilli	Leuconostocaceae	<0.01	0.04	0.08	0.1	0.2
Firmicutes	Bacilli	Streptococcaceae	0.4	0.2	0.3	**8.9**	**11.9**
Firmicutes	Clostridia	Clostridiaceae	6.0	<0.01	0.01	0.2	0.2
Firmicutes	Clostridia	Other^d^	<0.01	<0.01	0.02	0.4	0.7
Firmicutes	Erysipelotrichi	Erysipelotrichaceae	<0.01	0.05	0.05	0.04	0.7
Tenericutes	Mollicutes		<0.01	<0.01	<0.01	0.02	1.1
Proteobacteria	Gammaproteobacteria	Enterobacteriaceae	**61.1**	0.05	0.04	3.0	3.2
Unclassified			3.5	<0.01	<0.01	<0.01	0.2
Other			1.9	<0.01	<0.01	<0.01	0.1
*Total*			*99.3*	*99.9*	*99.9*	*99.7*	*98.6*

^a,b^ *Abbreviations*: *WA1* without antibiotic day 1; *WA5* without antibiotic day 5; *A5* with antibiotic day 5; *WA14* without antibiotic day 14; *A14* with antibiotic day 14

^c^average relative contribution of 8 pools consisting of 10 chicken

^d^Other is a limitation of the underlying bioinformatics tool to further classify this as a certain family

In **bold** are the two most dominant families per time-point

**Fig. 2:**
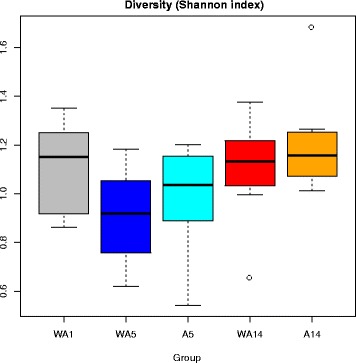
Diversity of luminal microbiota in jejunum of broilers for different experimental conditionsThe Shannon index (y-axis) was calculated for all five experimental conditions (WA1, WA5, A5, WA14, A14) (x-axis) based on the genus/species level. In total there are 5 treatment-day combinations consisting of up to 8 points, where each point represents 1 pool consisting of 10 chicken. At each time point eight measurements were performed, i.e. the spread depicted here is the average between pens. Abbreviations: WA1, without antibiotic day 1; WA5, without antibiotic day 5; A5, with antibiotic day 5; WA14, without antibiotic day 14; A14, with antibiotic day 14

**Fig. 3:**
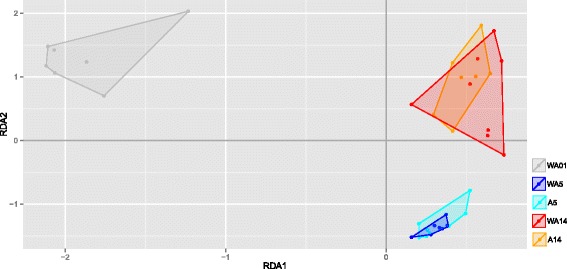
Redundancy analysis (RDA) of family level microbial groupsThe x-axis depicts explanatory axis 1 (RDA1) and y-axis depicts explanatory axis 2 (RDA2). Each condition is represented by a different colour (day 1, *grey*; day 5, where ‘WA’ is *blue* and ‘A’ is *cyan*; and day 14 where ‘WA’ is *red* and ‘A’ is *orange*). In total there are 5 treatment-day combinations consisting of up to 8 points, where each point represents 1 pool consisting of 10 chicken. The grey arrows represent the environmental variables as constraining variables (i.e. the different microbial groups). In total there are 5 treatment-day combinations consisting of up to 8 points (pools), where each point represents 1 pool consisting of 10 chicken. The following model was used as input for the RDA: y = Time + Treatment + Time* Treatment + error. Abbreviations: WA1, without antibiotic day 1; WA5, without antibiotic day 5; A5, with antibiotic day 5; WA14, without antibiotic day 14; A14, with antibiotic day 14

The microbiota diversity, calculated by the Shannon diversity index, was based on the genus/species level data. A decreasing trend in diversity was observed from day 1 (control) to day 5 (control, *p* = 0.08), whereas no significant difference between day 1 (control) to day 5 (antibiotic treated) (*p* = 0.32). From day 5 to day 14 an increasing trend in diversity was observed for both the control and antibiotic treated birds (*p* = 0.08 and *p* = 0.07, respectively) (Fig. 2). To investigate the microbiota composition as a whole, multivariate redundancy analysis (RDA) of the (approximate) family-level was performed, which showed a clear separation of time (days of age and time after antibiotic treatment). In addition, a high overlap was observed between the experimental treatments on each sampling day (Fig. 3). At the first axis (x-axis) 39% of the variance is explained and 10% of variance is explained at the second axis (y-axis). To test whether specific microbial families were significantly different between the treatment and control birds on a specific time-point, a Wilcoxon signed-rank test was employed. This resulted in eight significantly different family groups (*p* < 0.05) for day 5, and three significantly different family groups (*p* < 0.05) for day 14. However, when multiple testing correction was taken into account only three family groups remain for day 5, and for day 14 no family groups were left (Table 5).

**Table 5:** Statistical testing of family level microbial groups by Wilcoxon signed-rank test

Day	Phylum	Class	Family	*p*-value*	FDR
5	Firmicutes	Bacilli	Bacillaceae	<0.01	0.04
	Firmicutes	Bacilli	Carnobacteriaceae	<0.01	0.04
	Firmicutes	Bacilli	Leuconostocaceae	<0.01	0.04
	Actinobacteria	Actinobacteria	Nocardiopsaceae	0.02	0.11
	Firmicutes	Bacilli	Thermoactinomycetaceae	0.02	0.11
	Firmicutes	Clostridia	Ruminococcaceae	0.03	0.13
	Actinobacteria	Other	Other^a^	0.04	0.15
	Unclassified	Other	Other	0.05	0.15
14	Firmicutes	Bacilli	Enterococcaceae	0.01	0.24
	Firmicutes	Bacilli	Lactobacillaceae	0.03	0.24
	Firmicutes	Clostridia	Other	0.04	0.24

*Wilcoxon signed-rank test

^a^Other is a limitation of the underlying bioinformatics tool to further classify this as a certain family

### Transcriptomic analyses

**Fig. 4:**
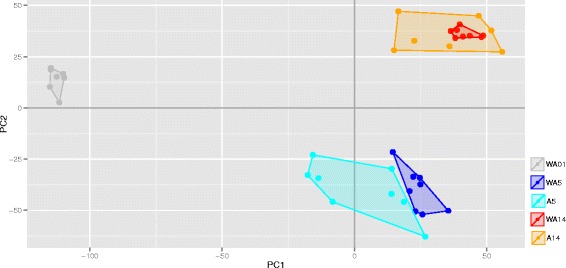
Principal components analysis (PCA) of complete intestinal transcriptomics dataThe x-axis depicts principal component 1 (PC1) and y-axis depicts principal component 1 (PC2). Each day is represented by a different symbol (day 1, *square*; day 5, *circle*; and day 14, *triangle*), and each treatment within a particular day by a colour (day 1, *grey*; day 5, where ‘WA’ is *blue* and ‘A’ is *cyan*; and day 14 where ‘WA’ is *red* and ‘A’ is *orange*). In total there are 5 treatment-day combinations consisting of up to 8 points, where each point represents 1 pool consisting of 10 chicken. Abbreviations: WA1, without antibiotic day 1; WA5, without antibiotic day 5; A5, with antibiotic day 5; WA14, without antibiotic day 14; A14, with antibiotic day 14

Principal Component Analysis (PCA) was performed to get insight into the variability in the jejunal transcriptomics data, taking into account the two treatment and three time-points. Only the first and second principal component were taken into account for both analyses, accounting for 37% and 15% of the variance, respectively. Figure 4 shows that clustering of the day/treatment groups occurred only on days and not on treatments. Furthermore, at this PCA level no within-days effects of the antibiotic treatment could be observed. To investigate the effect of the treatment in jejunum in more detail, an Analysis of Variance (ANOVA) was performed. All the probes (of which some with annotation) that were significant under p_adj_ < 0.01 were identified. Probes were also identified with a less stringent cut-off for the statistical testing p_adj_ < 0.05, but including an absolute Fold Change > 1 (Table 6). The annotated genes from the p_adj_ < 0.01 list were taken for further functional and enrichment analyses (Additional file 1). From these lists, both the significant up- and down-regulated genes were used as input for functional analyses which were performed by using methods within the DAVID software. This analysis resulted in multiple (range 11–172) gene clusters with a significant Enrichment Score (ES). The top 10 results from the DAVID functional annotation clustering are summarized in Table 7 for the comparison between treatments on day 5 and in Table 8 for the comparisons on day 14 (see also Additional file 2, for full analysis). In general, the enrichment scores for day 5 were higher compared to those of day 14. At day 5 the dominant terms of the down-regulated genes, i.e. lower expression in the antibiotic treated birds, were related to various immune processes, including ‘immune response-regulating signal transduction’, ‘Positive regulation of immune system process’, and’adaptive immune response’. The up-regulated gene clusters of day 5, i.e. higher expression in the antibiotic treated birds, mainly encoded for cellular processes, including ‘extracellular matrix’, ‘cell projection morphogenesis’, ‘regulation of cell development’, and ‘EGF-like domain’. At day 14 the down-regulated genes seemed to be involved in cellular processes, whereas the up-regulated genes did not show a coherent picture.

**Table 6:** Descriptive statistics of gene expression data of jejunum comparing antibiotic versus control on days 5 and 14

Comparison	A5-WA5 ^a,b^		A14-WA14	
Regulation	Down	Up	Down	Up
*Number of probes*				
p_adj_^c^ < 0.01	717	1156	457	354
p_adj_ < 0.05 and logFC^d^ > |1|	26	65	17	21
*Number of annotated genes*				
p_adj_ < 0.01	489	556	182	234
p_adj_ < 0.05 and logFC > |1|	18	18	8	3

^a,b^ *Abbreviations*: *WA5* without antibiotic day 5; *A5* with antibiotic day 5; *WA14* without antibiotic day 14; *A14*, with antibiotic day 14

^c^Adjusted *p*-value (False Discovery Rate)

^d^log Fold Change

**Table 7:** Functional annotation clustering (DAVID) of jejunum results (ES > 1.3) of the comparison antibiotic versus control on day 5 (p_adj_ < 0.01)

Down (lower in antibiotic treatment)		Up (higher in antibiotic treatment)	
ES^a^	General Term	ES	General Term
4.83	intracellular organelle lumen	7.86	extracellular matrix
4.77	protein transport/localization	5.25	triple helix (hydroxyproline,hydroxylysine)
3.26	domain: BTB/POZ-like	5.16	Collagen triple helix repeat (hydroxyproline,hydroxylysine)
3.09	macromolecule/protein catabolic process	4.47	cell projection morphogenesis (neuron, differentiation)
2.65	immune response-regulating signal transduction	3.66	Fibrillar collagen
2.39	nuclear envelope-ER network	3.56	regulation of cell development (neuronal)
2.33	Pos. regulation of immune system process	3.08	positive regulation of transcription/macromolecule
2.27	cellular protein localization	3.07	EGF-like domain
2.19	adaptive immune response	2.57	response to steroid hormone stimulus (cortico/glucocortico)
2.08	Protease/peptidase activity	2.57	thrombospondin-type (Laminin G)

^a^ES, Enrichment score

**Table 8:** Functional annotation clustering (DAVID) of jejunum results (ES > 1.3) of the comparison antibiotic versus control on day 14 (p_adj_ < 0.01)

Down (lower in antibiotic treatment)		Up (higher in antibiotic treatment)	
ES ^a^	General Term	ES	General Term
2.49	positive regulation of biosynthetic process/transcription	4.5	organelle lumen (intracellular)
2.00	epithelium morphogenesis/development	2.38	transit peptide/Mitochondrion
1.60	macromolecule/protein catabolic process	1.84	sterol/steroid biosynthesis
1.48	intracellular organelle lumen	1.68	Heat shock protein (DnaJ)
1.47	blood vessel development	1.53	RNA recognition motif (RNP-1)
		1.51	translation initiation factor activity
		1.48	(negative) regulation of lipid storage
		1.43	Multiple Signalling Pathways (EPO/IGF1/IL6/TPO/IL2/PDGF/EGF)
		1.39	cellular protein localization/targeting
		1.32	zinc-binding (LIM domain)

^a^ ES, Enrichment score

### Immunohistochemistry

**Fig. 5:**
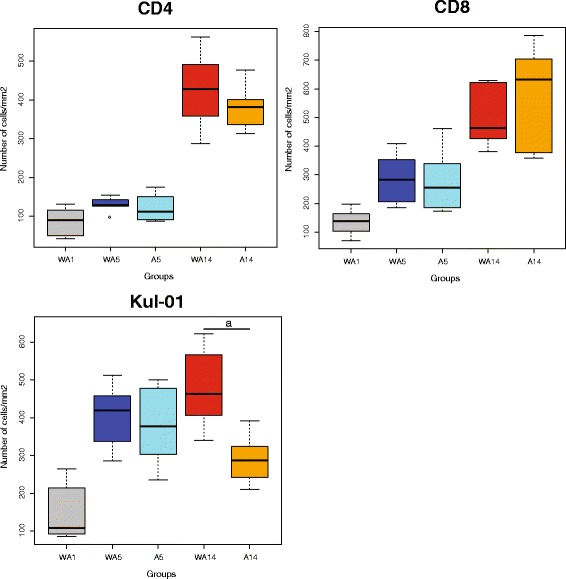
Immunohistochemistry (IHC) staining of immune cells for the different treatments in time. In all graphs, the horizontal axis depicts the experimental condition (treatment and time) and the vertical axis the number of cells per square millimetre of jejunal tissue. Left upper panel depicts CD4^+^ cells, right upper panel shows the CD8^+^ cells, and the lower left panel represents the KUL01^+^ cells. In total there are 5 treatment-day combinations consisting of up to 8 points, where each point represents 1 pool consisting of 10 chicken. At each time point eight measurements were performed, i.e. the spread depicted here is the average between pens. Abbreviations: WA1, without antibiotic day 1; WA5, without antibiotic day 5; A5, with antibiotic day 5; WA14, without antibiotic day 14; A14, with antibiotic day 14

To investigate whether the differences observed at the gene expression level at day 5 were translated into a difference in the number and/or identity of immune cells in the gut mucosal tissue, we analysed intestinal tissue sections for the presence of macrophage(-like) cells, CD4^+^ and CD8^+^ cells. Macrophage(-like) cells have a strong link to innate immunity, whereas CD4^+^ and CD8^+^ have a strong link to adaptive immunity. All measurements were performed in jejunal mucosa tissues at days 1, 5, and 14 (Fig. 5). The development of the innate and adaptive immune system over time was shown by the increasing number of cells per consecutive time-points. Significant differences were observed between day 1 and 5 for CD4^+^ (*p* < 0.01), CD8^+^ (*p* < 0.001), and KUL01^+^ (*p* < 0.001). Between day 5 and 14, only CD4^+^ (*p* < 0.001) and CD8^+^ (*p* < 0.001) were significantly different. No significant difference was observed on days 5 and 14 when testing for the effect of the antibiotic treatment in either CD4^+^ or CD8^+^ cells. However, for KUL01^+^ cells (monocytes/macrophages) a significant decrease in the treatment group (*p* < 0.001) was observed on day 14.

## Discussion

It is known that perturbation of intestinal microbial colonization by antibiotic usage during early life influences immune development, e.g. children who received antibiotics have higher incidence of allergy and asthma [31–33]. Besides these seemingly negative effects on immune development, one can imagine that it may also be possible to positively modulate immune development by providing specific diets or feed additives that influence the microbial colonization of the gut. In this paper we provide evidence that a 24 h oral antibiotic treatment during early life of broilers has a limited effect on the microbial composition at day 5 after treatment, has a significant effect on the intestinal gene expression profile later in life (days 5 and 14), and a significant effect on the number of macrophages (day 14) in intestinal mucosal tissue. We expect the antibiotic effect to be greater than the environmental effect. However, we do expect that the housing system, i.e. floor pens (this study) or cages, could have an effect on the ultimate microbiota composition.

In this study we investigated the local impact of an antibiotic treatment in the gut. Therefore we sampled jejunum for determining both gene expression and microbiota profiling. The jejunum is important for the absorption of nutrients and harbours immune cells that are important for monitoring luminal content (e.g. antigens). However, one must realize that the jejunum forms part of a larger complex ecosystem, the gastro-intestinal tract. Antibiotics-induced changes may therefore also occur in up- or downstream parts of the gastro-intestinal tract, for example in the ileum or caecum. Furthermore, the observed antibiotic-induced local changes may also trigger differences in immune programming systemically. However, because of the lack of data, we cannot conclude whether this occurs.

### Microbial colonization and the effect of a short antibiotic treatment

A few studies have been performed addressing the temporal development of the most important colonizers of the small intestine or caecum of chickens [3, 4, 34–36]. Here we observed that at day 1 the *Enterobacteriaceae* and *Enterococcaceae* families were most abundant. At day 5 the gut ecosystem shifted towards *Lactobacillaceae* being most dominantly present followed by *Enterococcaceae*. At day 14 *Lactobacillaceae* were still the most dominant family and *Streptococcaceae* were second most abundant. This shows the generic succession of bacterial families in the chicken gut over time, regardless of the antibiotic treatment perturbation.

The diversity, as measured by the Shannon index, showed a decreasing trend from day 1 (control) to day 5 (control) or no significant difference between day 1 (control) to day 5 (antibiotic treated). This was observed in both control and antibiotic treated chickens and may be due to the change in environmental conditions. At day 1, chickens were transported by truck from the hatchery to the experimental farm, this change of environment could have accommodated stress which is known to affect the gut microbiota and therefore the microbial diversity [37, 38]. Another explanation might be that the decreasing trend in microbiota diversity is due to the lack of feed intake during transport. An increasing trend of the diversity from day 5 to 14 was observed for both the control and antibiotic treated birds, this was expected because, in general, the whole gut system develops towards a highly diverse and more stable system, already partly reflecting the ‘adult’ type microbiota [39–41].

When comparing the antibiotic treated chickens with their respective controls, we only observed a numerical increased diversity in the antibiotic group at day 5, that was not notable anymore at day 14. This early life antibiotic-driven increase in diversity was similar to previous observation in pigs, showing that the microbial diversity slightly increases and the microbial community structure becomes more chaotic [11, 15, 19]. Increase of diversity in an early life colonizing gut system generates more chaos and is assumed to be detrimental for immune development and therefore “bad” [10]. Whereas increase of diversity in developed “stable” gut systems is assumed to be associated with improved resilience of that system.

When comparing the microbiota composition of the antibiotic treated and control chickens, a high overlap was observed for both day 5 and 14.. We expected long-lasting changes based upon previous findings [15, 18, 42–44], however in this experiment the gut microbiota ecosystem apparently developed towards a steady state in 3 days after the antibiotic treatment.

The impact of the antibiotic treatment could be observed on the bacterial family level where minor changes in average relative contribution (ARC) occurred at day 5 and major changes on day 14. *Lactobacillaceae* were more abundant at day 14 in controls compared to the antibiotic treated chickens (*p* = 0.03, FDR 0.24), 82.2% and 70.5% respectively. This suggests that the early antibiotic treatment affected the microbial colonization and composition/diversity over a long period of time. This could be due to the antibiotic treatment per se or to a shifting of the microbial ecosystem towards a different steady state (homeostasis). Lactobacilli were used as probiotics in chicken to improve intestinal health [45–47] and are reported to be involved in competitive exclusion of pathogens [48–50], however it has also been shown that they may have a negative effect due to deconjugation of bile acids [51, 52]. This suggests that, in general, a high abundance of Lactobacilli is favourable for intestinal health. Since the abundance of *Lactobacillaceae* was decreased in the antibiotic group, this also implies that a perturbation with amoxicillin may have a negative effect on (gut) health by the principle of competitive exclusion. In our study, we observed a higher abundance of lactobacilli, higher expression of immune related genes, and a higher abundance of macrophage like cells, suggesting that the latter may also contribute to improved health. The higher abundance of *Lactobacillaceae* did, however, not translate into a measurable change in body weight or feed conversion ratios, although the number of animals (*n* = 8 pens per treatment group) might be too low to draw firm conclusions on this aspect. At day 14, a higher numerical abundance of *Enterococcaceae* was observed in the antibiotic treated group (9.9% ARC) compared to the control (4.9% ARC) chickens. This could be due to the presence of intrinsic antibiotic resistance mechanisms [53] as have been described for *Enterococcus faecium* and *Enterococcus faecalis*. Unfortunately, it was not possible to check whether the Enterococci have antibiotic resistance gene cassettes present.

### Functional genomics approach identifies (dis)similarities between antibiotic treated and control chickens

The antibiotic treated chickens showed downregulation of genes involved in immune related processes at day 5 and (generic) metabolic processes at day 14. Whereas, upregulation of genes associated to cell structure/cell cycle and developmental processes was observed in the antibiotic treated chickens at day 5. Similar observations have been described for piglets after an antibiotic treatment during early life. These antibiotic treated piglets showed decreased mucosal gene expression profiles and a subset of the down-regulated genes are involved in immune related processes [11, 15, 19]. Our gene expression data suggest that a perturbed microbial colonization in the chicken gut leads to downregulation of immune related genes (including the following genes BCL10, PSEN1, LYN, PSEN2, TLR4, TLR6, TLR7, C1QB, and C1S) and an upregulation of genes linked to cell development and intestinal barrier function (including FMOD, LTBP2, HMCN1, WNT3, SMOC1, AGRN, ENTPD2, MUC2, ZP1, NTN1, ADAMTS5, several genes involved in adhesion, and many collagen genes). Functional analysis of the down-regulated immune genes pointed towards a decrease or delay in the development of cell-mediated immunity. Because under normal conditions cell-mediated immunity develops immediately after hatch and maturation occurs primarily in the first week [13, 14], this decrease or delay maybe directly linked to the microbiota-driven programming of the immune system. The barrier function of the intestinal epithelium is the first line of defence against intruders. Dysfunction of the intestinal barrier leads to loss of epithelial integrity and a higher risk for multiple gastrointestinal diseases [54–57]. Altogether, the gene expression data suggested that due to the antibiotic treatment, the developmental “priorities” of the gut have shifted away from cell-mediated immune development in favour of strengthening the gut barrier functions. This strategy of coping with a perturbation during early life could be most cost effective for the birds, because strengthening the intestinal barrier results in less short-time risks for the invasion of (pathogenic) microorganism. For the long term this strategy may result in a reduced immune competence of the birds.

When taken both the microbiota and transcriptomics data together, the presented data suggest that an antibiotic treatment during early life only causes limited changes in microbiota diversity and/or composition. However, these limited and temporal changes may exert a significant influence on immune programming. This may be due to the fact that jejunum was chosen as the tissue of interest, which has a less complex microbiota composition and diversity compared to caecum, Shannon index 2.5 for jejunum and 5.5 for caecum [58]. We still need more knowledge about this early life phase to fully understand the gut (eco)system and its implication towards the development and programming of the immune system. Nevertheless, this is a first step in identifying key components, e.g. microbial families or species, which are involved in early life gut development.

### Differences in immune cell populations due to the antibiotic treatment

Development of the adaptive immune system in broilers occurs in the first weeks of life [12–14]. Both CD4^+^ and CD8^+^ cells increase significantly in number in the small intestine from day 1 to 5 and day 5 to 14, whereas the macrophage-like cells only significantly increase in number at day 5. Here, we only investigated the entire CD4^+^ population, which limits the interpretation towards the ratio between Th1 and Th2 cells. This ratio is important since it balances the systems between inflammation and antibody production [59]. To our knowledge, the (in)direct effect of amoxicillin on immune cells in healthy animals is not well described yet, although it has been described that an beta lactam antibiotic influences cytokine expression but not the number of immune cells [60]. Our gene expression data already suggested an effect of amoxicillin on immune cells, because we observed differential expression of genes like HLA-DRB1 (alias DR1), GSTT1, CYP19A1, CXCL8 (alias IL8), and CRP (Additional file 1). All these genes are involved in different aspects of cell-based immunity [61–65]. These genes are involved in a range of processes including antigen processing and presentation, natural killer cell mediated cytotoxicity, and hematopoietic cell lineage. It is tempting to speculate that the differences in these gene expression profiles translate into differences at the cellular level. With regard to macrophages this was indeed the case. The number of macrophages was significantly lower in antibiotic treated chickens on day 14 compared to control chickens, whereas only a decreasing trend in the number of macrophage-like was observed on day 5. We speculate that the downregulation of genes involved in immune processes, as observed at day 5 in the antibiotic treated chickens, has a direct effect on the number of macrophages in later life. Another possibility is that due to the augmented barrier function, chemo attraction and/or influx of macrophages are reduced under such conditions. Although we do not have any information on the activity of the macrophages, the consequence of our observations could be that antibiotic treated chickens have a reduced or altered innate immune competence. Since innate and adaptive immunity are intertwined, this may also affect adaptive immune responses later in life. The observed numerical decrease in the number of CD4^+^ cells in the antibiotic treated birds compared to control birds, is in agreement with this. The number of CD8^+^ cells was not found to be affected by the antibiotic treatment, suggesting a limited impact of the antibiotic on this structural component of the adaptive immune system.

## Conclusion

Short term oral perturbation with an antibiotic during early life of chickens affects microbial colonization and intestinal immune development over a period of 2 weeks. This was shown as a trend at the microbiota level (composition and diversity), but significant at the gene expression level in the mucosa of the small intestine. Furthermore, we validated that the observed changes at the gene expression level most probably lead to alterations at the cellular immune level, i.e. changes in the number of macrophage-like cells. Our data support the assumption that early life colonization of the gut by microbiota is an important driver of immune development and/or immune programming, as has been found for other (mammalian) species. However, we could not rule out direct effects of amoxicillin on immunity. We conclude that it might be worthwhile to explore the capabilities of a variety of early life dietary and/or management factors modulate (indirectly via the microbiota) immune competence development of broilers. Furthermore, our data point towards potential microbial, gene expression-based, and cell-based indicators that might be used by animal nutrition industries for the development of innovative products to optimize immune competence in broilers. Finally, our data provide some preliminary insight into the mechanisms underlying the increased risk for disease development (predisposition of pathogenic bacterial species) associated with early life usage of antibiotics. This usage leads to decreased expression of genes involved in immunological processes at day 5 and subsequently lower number of macrophage-like cells at day 14 in jejunum.

This work shows that it is possible to modulate the microbiota via antibiotics with a negative impact on immune development. Therefore, it may also be possible to modulate the early life colonization of ‘beneficial’ microbiota by the application of innovative dietary-based or management measures. In this context it is worth mentioning that this study provides a valuable resource for the identification of bacterial families of possible new probiotic starter strains and/or targets for new early life prebiotics that may be developed in the future. Current pre- and probiotics products, although also sometimes given during early life, are mainly based on microbiota and knowledge gained from adult birds [47, 66–68]. Probiotic starter products based on the knowledge provided herein, may be used to optimize early life immune development and immune programming of poultry with the ultimate aim to improve poultry’s immune competence.

## Additional files

Additional file 1: Full analysis of differential expressed genes. The Additional File (presented as.xlsx) contains multiple worksheets, where the first worksheet describes the data present in the other four worksheets. Worksheets 2 and 3 contain the full LIMMA results for respectively the contrast A5-WA5 and A14-WA14. Whereas worksheets 4 contains the gene names (*p* < 0.01) which were used a input for DAVID functional annotation clustering. Worksheet 5 contains probes (genes) which were below adjusted *p*-value of 0.05 and had a Fold Change (FC) above 1 or below -1. (XLSX 4618 kb)
Additional file 2: Full analysis of functional annotation clustering. The Additional File (presented as.xlsx) contains multiple worksheets, where the first worksheet describes the data present in the other five worksheets. Worksheets 2–5 contain the full DAVID functional annotation clustering results for the contrast A5-WA5 (up- and down-regulated genes) and A14-WA14 (up- and down-regulated genes). Whereas worksheets 4 contains the gene names (*p* < 0.01) which were used a input for DAVID functional annotation clustering. Worksheet 5 contains probes (genes) which were below adjusted *p*-value of 0.05 and had a Fold Change (FC) above 1 or below -1. (XLSX 5707 kb)

## Abbreviations

ANOVA: Analysis of Variance
ARC: Average relative contribution
DAVID: Database for Annotation, Visualization and Integrated Discovery
ES: Enrichment score
FAC: Functional annotation clustering
FCR: Feed conversion ratio
FDR: False discovery rate
LIMMA: Linear models for microarray data
OTU: Operational taxonomic unit
PCA: Principal Component Analysis
QIIME: Quantitative Insights Into Microbial Ecology
RDA: Redundancy analysis
